# Liver transcriptome profiling and functional analysis of intrauterine growth restriction (IUGR) piglets reveals a genetic correction and sexual-dimorphic gene expression during postnatal development

**DOI:** 10.1186/s12864-020-07094-9

**Published:** 2020-10-08

**Authors:** Hongmei Gao, Longchao Zhang, Ligang Wang, Xin Liu, Xinhua Hou, Fuping Zhao, Hua Yan, Lixian Wang

**Affiliations:** Institute of Animal Science, Chinese Academy of Agricultural Sciences, Beijing, 100193 P. R. China

**Keywords:** Intrauterine growth restriction (IUGR), Piglets, Liver, Transcriptome, Sexual dimorphism

## Abstract

**Background:**

Intrauterine growth restriction (IUGR) remains a major problem associated with swine production. Thus, understanding the physiological changes of postnatal IUGR piglets would aid in improving growth performance. Moreover, liver metabolism plays an important role in the growth and survival of neonatal piglets.

**Results:**

By profiling the transcriptome of liver samples on postnatal Days 1, 7, and 28, our study focused on characterizing the growth, function, and metabolism in the liver of IUGR neonatal piglets. Our study demonstrates that the livers of IUGR piglets were associated with a series of complications, including inflammatory stress and immune dysregulation; cytoskeleton and membrane structure disorganization; dysregulated transcription events; and abnormal glucocorticoid metabolism. In addition, the abnormal liver function index in the serum [alanine aminotransferase (ALT), aspartate aminotransferase (AST), and total protein (TP)], coupled with hepatic pathological and ultrastructural morphological changes are indicative of liver damage and dysfunction in IUGR piglets. Moreover, these results reveal the sex-biased developmental dynamics between male and female IUGR piglets, and that male IUGR piglets may be more sensitive to disrupted metabolic homeostasis.

**Conclusions:**

These observations provide a detailed reference for understanding the mechanisms and characterizations of IUGR liver functions, and suggest that the potential strategies for improving the survival and growth performance of IUGR offspring should consider the balance between postnatal catch-up growth and adverse metabolic consequences. In particular, sex-specific intervention strategies should be considered for both female and male IUGR piglets.

## Background

Intrauterine growth restriction (IUGR) is typically defined as mammalian neonates with a low birth weight due to intrauterine crowding and placental insufficiency, resulting in impaired fetal or postnatal growth and development [1]. Among livestock species, pigs exhibit the most frequent occurrence of IUGR [2]. Moreover, IUGR piglets have been shown to be correlated with high morbidity and mortality, stunted growth, as well as poor carcass quality [1]. Great efforts have been made to minimize the negative effects of IUGR, and some investigations have shown that dietary nutrient supplementation can improve the survival and growth performance of IUGR piglets (e.g.*,* mid-chain triglycerides [3], choline [4], arginine [5], and dimethylglycine sodium salt [6]). However, the underlying mechanisms of nutrient utilization in IUGR piglets were not well defined, and it is difficult to take effective measures to maximize the performance of IUGR piglets.

The liver plays a vital role in nutrient utilization and metabolism, as well as in endocrine and immune homeostasis. Epidemiological studies have indicated that IUGR neonatal livers were accompanied by metabolic disorders during the postnatal period (e.g.*,* disruption in mitochondrial oxidative phosphorylation and energy metabolism [7–9]). Additionally, the IUGR neonates have been shown to be highly prone to developing metabolic syndrome (e.g.*,* obesity and diabetes) due to the increasing hepatic gluconeogenic capacity and impairing β-cell function [10, 11]. However, the precise mechanisms associated with IUGR piglet liver function remain poorly understood.

High-throughput methods have been widely applied to understand both the physiological and pathological characteristics in the liver of various species [12–14]. In this study, we compared the liver transcriptomes between IUGR and normal neonatal piglets from Day 1 to Day 7, to the weaning day (Day 28) using whole-genome transcriptional sequencing, to gain insight into the dynamics of metabolism, growth, and development in IUGR piglets. The results demonstrate that the altered glucocorticoid signaling pathway in IUGR newborn piglets may lead to immune deficiency and inflammation in the liver. In addition, for the first time, we have reported that IUGR affects liver function and metabolism in a sex-biased manner. Moreover, sexual dimorphism can be detected as early as postnatal Day 1. This also suggested that a sex-biased intervention strategy for IUGR should be specific to male or female IUGR piglets.

## Results

### Differences in the growth performance between the IUGR and normal body weight (NBW) piglets

**Fig. 1:**
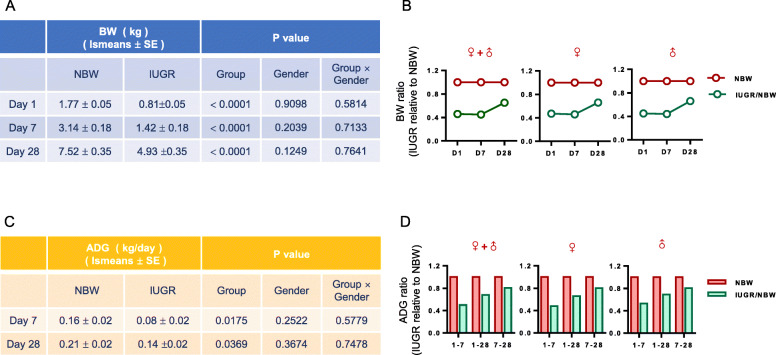
Growth performance between IUGR and NBW piglets. **a** Body weight in the IUGR and NBW piglets; BW, body weight. The data are expressed as the lsmeans ± SE, and the associated *P* value was presented to indicate statistical significance between the IUGR and NBW groups. **b** Body weight of the IUGR piglets relative to that of the NBW piglets. **c** The average daily gain in the IUGR and NBW piglets. ADG, average daily gain. **d** Average daily gain of IUGR piglets relative to that of the NBW piglets

In this study, the body weight of all piglets was summarized in Fig. 1a. The initial body weight of the IUGR neonates was significantly lower than that of the NBW on Day 1 as expected (*P* < 0.01). However, the body weight of the IUGR piglets was consistently lower than that of the NBW on Day 7 and Day 28 (P < 0.01). By calculating the relative body weight of the IUGR piglets to NBW piglets, the results showed that the body weight ratios were 45, 44, and 66% on Days 1, 7, and 28, respectively (Fig. 1b). It was noteworthy that the gaps in body weight between the IUGR and NBW piglets was reduced on Day 28 compared with that on Day 1 and Day7, which implies a catch-up growth compensation in IUGR piglets.

Furthermore, in line with the decreased body weight difference between the IUGR and NBW piglets, growth compensation was also supported by the increasing ADG ratio of the IUGR piglets throughout the postnatal period (Fig. 1c and d). In addition, no significant sexual-dimorphic effects on the growth performance of the body weight and ADG were observed between the IUGR and NBW piglets at each time point.

### General profiling of DEGs between the IUGR and NBW piglets

**Fig. 2:**
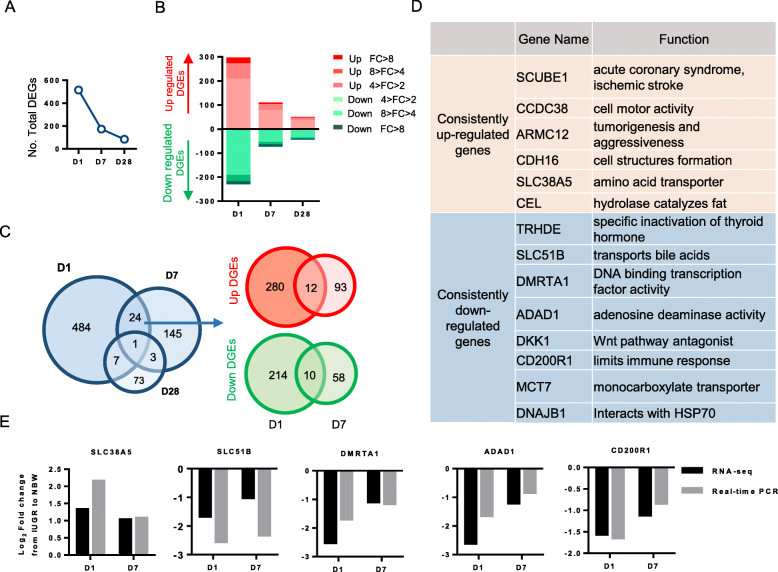
General functional profiling of the DEGs (*P* < 0.05) whose expression significantly changed (fold-changes (FC) > 2 or < 0.5) between the IUGR and NBW piglets. **a** The total number of differentially expressed genes (DEGs) on Days 1 (D1), 7 (D7), and 28 (D28). **b** Distribution of DEGs with different fold-changes on D1, D7, and D28. Different fold-changes are represented by different colors. The number of DEGs from each subcategory are indicated on the right. **c** Venn diagrams of consistently dysregulated DEGs on D1, D7, and D28 (left panel), as well as upregulated (right panel, red) and downregulated (right panel, green) DEGs from postnatal Days 1 to 7. **d** The tables show the major functions of DEGs that are consistently upregulated or downregulated from postnatal Day 1 to 7. **e** Comparison of the real-time qPCR and RNA-Seq results of the DEGs

Transcriptome sequencing was performed using a total of 42 liver samples from the IUGR and NBW piglets on Days 1, 7, and 28, respectively [Day 1: IUGR *n* = 8 (4 females and 4 males) vs NBW n = 8 (4 females and 4 males); Day 7: IUGR *n* = 7 (4 females and 3 males) vs NBW n = 7 (4 females and 3 males); Day 28: IUGR *n* = 6 (3 females and 3 males) vs NBW n = 6 (3 females and 3 males)]. Approximately 20,000 transcripts were detected in each sample. Compared with NBW, the liver of IUGR piglets contained 516 differentially expressed genes (DEGs) on Day 1 (*P* < 0.05; FC > 2 or < 0.5). Of these, 292 were up-regulated and 224 were down-regulated. On Day 7, 173 DEGs were screened out, 105 of which were upregulated and 68 were downregulated. Notably, the number of DEGs decreased along with the postnatal period, and only 84 DEGs were screened out on Day 28. At each time point, the mildly altered DEGs (4 > FC > 2 or 0.5 > FC > 0.25) accounted for the largest proportion of DEGs (Fig. 2a and b; Supplementary file: Table S1-S3). These results suggested that the altered gene expression profiles in the IUGR piglet livers could be attenuated with postnatal development.

In addition, a Venn diagram was used to screen the consistently dysregulated DEGs during the postnatal stage. The results showed that an extremely small number of DEGs were consistently regulated between each time point. Only one DEG was consistently dysregulated throughout the entire postnatal period in the IUGR piglets. There were 24 DEGs that were consistently dysregulated from Days 1 to 7, 3 DEGs were consistently dysregulated from Days 7 to 28. There were 484, 145, and 73 DEGs specifically dysregulated on Days 1, 7, and 28, respectively. The large proportion of stage-specific DEGs at each time point suggested that disordered liver functions or development are highly dynamic in IUGR piglets. Despite this finding, 12 and 10 DEGs were consistently up- and down-regulated from postnatal Days 1 to 7 (Fig. 2c). These DEGs were involved in multiple cellular processes, including inflammatory immunity (*SCUBE1* and *CD200R1*), nutrient transport (*SLC38A5*, *SLC51B*, and *MCT7*), and cellular proliferation and migration (*CCDC38*, *ARMC12*, and *CDH16*) (Fig. 2d). Five of these DEGs (*SLC38A5*, *SLC51B*, *DMRTA1*, *ADAD1*, and *CD200R1*) that were involved in important biological processes and functions, were further detected using real-time qPCR to validate the reliability of the RNA-Seq analysis (Fig. 2e).

### Detailed functional profiles of the DEGs between the IUGR and NBW piglets

**Fig. 3:**
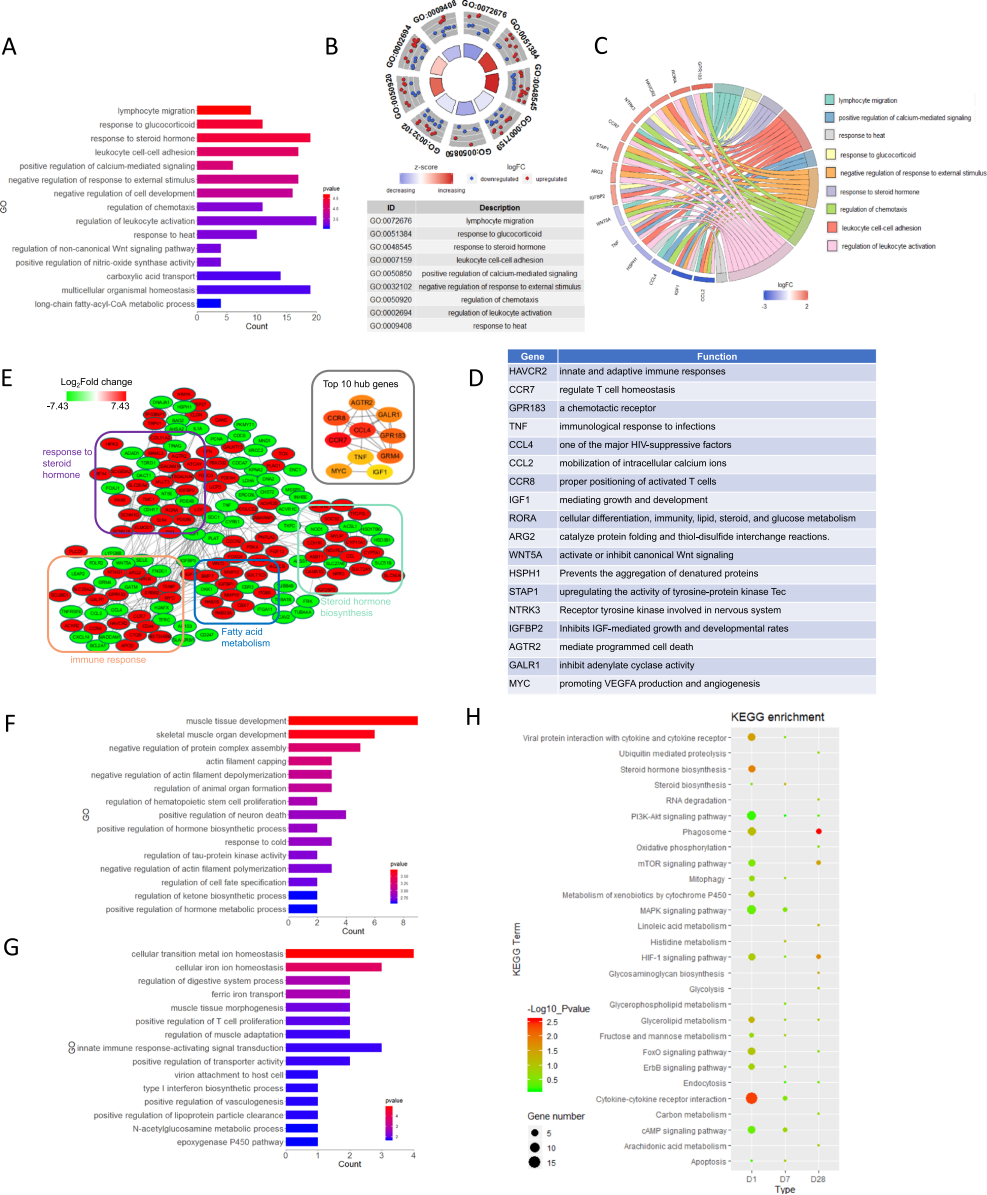
Detailed functional profiling of the DEGs whose expression significantly changed (P < 0.05, FC > 2 or < 0.5) between the IUGR and NBW piglets. **a** Classification of GO terms based on the functional annotation of BP enriched in the IUGR piglets on Day 1. The ordinate represents the GO item, the abscissa represents the number of enriched DEGs corresponding to each term, and the color column represents the enrichment score (defined as -Log10 *P*-value). **b** The GOCircle plot of IUGR piglets on Day 1. The outer circle shows a scatter plot for each term of the logFC of the assigned genes. The red circles indicate the upregulated genes and the blue circles indicate the down-regulated genes by default. **c** The GOChord plot of the IUGR piglets on Day 1. The DEGs that were assigned to at least three process terms were selected. **d** The tables show the major functions of the DEGs that were selected in the IUGR piglets on Day 1. **e** The protein-protein interaction network of the DEGs in the IUGR piglets on Day 1. The red nodes indicate gene upregulation and the green nodes indicate downregulation in IUGR piglets. Fold changes (FC) in expression are expressed as log2 (FC) values. **f** GO enrichment analysis of the DEGs of BP enriched in the IUGR piglets on Day 7. **g** GO enrichment analysis of the DEGs of BP enriched in the IUGR piglets on Day 28. **h** Enriched KEGG pathways (Top 15) for the DEGs that were significantly altered in the IUGR piglets during postnatal development

The following functional analyses were based on Gene Ontology (GO) for the dynamically altered DEGs between the IUGR and NBW piglets to explore the potential physiological changes in the IUGR liver. GO classification of the biological processes (BP) showed that the dysregulated DEGs were most significantly enriched in the hepatic immune response on Day 1, including ‘lymphocyte migration’, ‘leukocyte cell-cell adhesion’, ‘regulation of chemotaxis’, and ‘regulation of leukocyte activation’ (Fig. 3a). These findings suggest that the liver of IUGR piglets may suffer from immune-related stress. DEGs were also clustered in items, such as ‘response to glucocorticoid’ and ‘response to steroid hormone’, which may imply a disordered steroid hormone metabolism and response. It is important to note that most of the DEGs related to immune regulation were down-regulated, whereas those related to sterol hormone regulation were up-regulated through GOCircle plot analysis (Fig. 3b). We further focused on these DEGs, and the GOChord plot was performed to select the DEGs, which were assigned to at least three BP terms (Fig. 3c). Among these, *GPR183*, *STAP1*, *HAVCR2*, *CCR7*, *TNF*, *CCL4*, *WNT5A*, and *CCL2* were all involved in the innate and adaptive immune response and homeostasis, whereas *IGF1*, *IGFBP2*, *RORA*, *AGTR2*, *NTRK3*, and *HSPH1* were related to cellular growth, differentiation, and developmental regulation (Fig. 3d). To further investigate the functional relationship among the DEGs on Day 1, the protein-protein interaction (PPI) was constructed using the STRING database. The interconnected DEGs were also clustered in the subnetwork of steroid hormone biosynthesis and regulation, fatty acid metabolism, and immune response (Fig. 3e). Next, the node genes of the DEG network were ranked by the CytoHubba, and the top 10 hub genes and related functions were presented. These genes contained *TNF*, chemokines (*CCL4*), and their receptors (*CCR7* and *CCR8*), which can cause inflammation. It also contained genes from the G protein-coupled receptor family (*GPR183*, *GRM4*, *GALR1*, and *AGTR2*), which regulated G protein activity in the liver (Fig. 3d). Some of the screened DEGs were overlapping in the GOChord and CytoHubbar analysis, implying the importance of these genes in determining the phenotype of IUGR piglets.

Next, we performed a detailed analysis of the DEGs on Day 7. The majority of the DEGs were enriched in the regulation of actin filament depolymerization and polymerization processes. DEGs in these terms were primarily involved in the assembly of the actin filament network and maintenance of the actin skeleton (*ADD2*, *KIAA1211*, and *SPTB*). Moreover, the DEGs were also concentrated in the muscle tissue growth (*DKK1*, *EGR1*, *EGR2*, *FOS*, *KEL*, and *SHOX2*), as well as hormone biosynthesis and metabolism processes (*ADM* and *EGR1*) (Fig. 3f). These indicate that the dysregulated DEGs may affect the cytoskeleton reorganization in the IUGR liver tissue on Day 7. The DEGs on Day 28 were analyzed in the same manner, which were primarily enriched in the ‘cellular transition metal ion homeostasis’ process, including *ATP6V1G1*, *HAMP*, *SLC30A4*, and *TFRC*. Of these, both *HAMP* and *TFRC* regulated the maintenance of ion homeostasis, and *SLC30A4* exerted zinc transmembrane transporter activity. Dysregulation of transition metal ion homeostasis may be the molecular basis for the abnormal physiological characteristics of IUGR piglets. At the same time, these DEGs contained *CD209*, *TLR8*, and *UBE2D2*, which were clustered in inflammatory entries (e.g., ‘positive regulation of T cell proliferation’, ‘innate immune response-activating signal transduction’, and ‘type I interferon biosynthetic process’). All of these entries may be suggestive of an abnormal state of immune stress in IUGR piglets (Fig. 3g).

Finally, a KEGG analysis was performed to determine the pathways that participate in the disordered functions exhibited in the livers of the IUGR piglets. The PI3K-AKT signaling pathway, glycerolipid metabolism, and the HIF-1 signaling pathway were significantly enriched consistently during the postnatal period. Moreover, the cAMP signaling pathway, cytokine-cytokine receptor interaction, phagosome, MAPK signaling pathway, and steroid hormone biosynthesis were also enriched (Fig. 3h). These enrichment pathways fully revealed the pathophysiological status of the IUGR piglets. Moreover, the number and significance of the enriched pathways also supported the concept that disordered state of IUGR appeared to be alleviated during postnatal development.

### Analysis of serum biochemical parameters and liver histology between the IUGR and NBW piglets

**Fig. 4:**
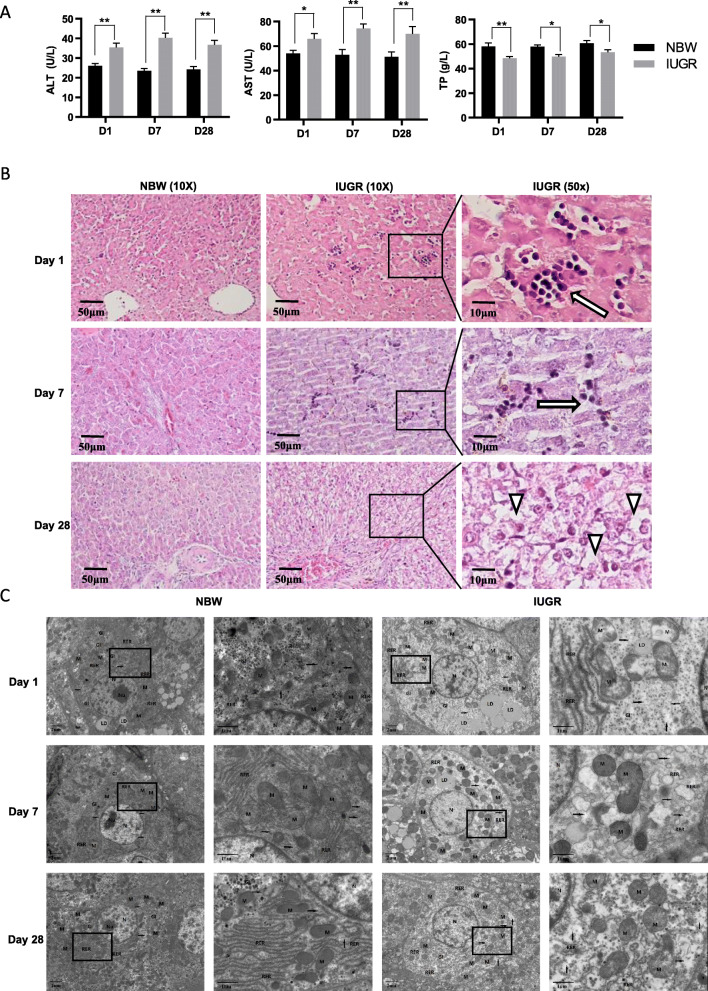
Functional detection of the livers between IUGR and NBW piglets. **a** The serum level of ALT, AST, and TP in the IUGR piglets compared with NBW (* *P* < 0.05; ** *P* < 0.01). **b** Light microscopy of the liver tissue between the NBW and IUGR groups on Days 1, 7, and 28 for Hematoxylin eosin (H&E) staining. indicates lymphocyte infiltration, and indicates vacuolization. **c** Representative transmission electron micrographs of the liver sections from NBW and IUGR groups on Days 1, 7, and 28. N: nucleus; Nu: nucleolus; M: mitochondria; RER: rough endoplasmic reticulum; Gl: glycogen; LD: lipid droplets; black arrows: cytoplasmic material loss

Given that the DEGs between IUGR and NBW piglets were related to the abnormal immune response, we next compared the liver function index between the IUGR and NBW piglets to assess the potential impact of immune stress on the liver damage in IUGR piglets. The liver function indexes in the IUGR piglets changed significantly, as the serum alanine aminotransferase (ALT) and aspartate aminotransferase (AST) activity in the IUGR piglets was significantly higher than that in the NBW piglets at all of the time points. Moreover, the total protein (TP) content, a biomarker of the inflammatory status in the liver, was found to be significantly lower in the IUGR piglets than that in the NBW piglets (Fig. 4a), which predicted the inflammatory status in the livers of the IUGR piglets.

We subsequently detected the hepatic pathological sections in IUGR piglets. Compared with the NBW piglets, the IUGR piglets displayed marked inflammatory lymphocytic infiltration in the hepatic lobules at different time points. Additionally, apparent vacuolar and severe structural damage appeared in the IUGR hepatocytes on Day 28 (Fig. 4b). These results further confirm the existence of liver injury in IUGR piglets.

In addition, a comparison of the ultrastructural morphology of the liver between IUGR and NBW piglets was evaluated using transmission electron microscopy (TEM). In the present study, ultrastructural pathological lesions were observed in the hepatocytes of IUGR piglets. Striking structural alterations were identified in the IUGR piglets, including vacuolar dilatation of the cytoplasm, loss of cytoplasmic material and degeneration of hepatocyte organelles, especially in the mitochondria and endoplasmic reticulum. These observations indicated that the mitochondria were swollen, round-shaped, and the mitochondrial cristae were disrupted. Furthermore, discontinuous endoplasmic reticulum cisternae were also observed among the hepatocytes in IUGR piglets at each time point. Whereas a normal histological appearance with well-organized organelles was observed in the liver sections of the NBW piglets (Fig. 4c). These results further support that ultrastructural cytoskeleton is disrupted in hepatocytes of IUGR piglets.

### Sexual-dimorphic effects on the liver expression patterns between the IUGR and NBW piglets

**Fig. 5:**
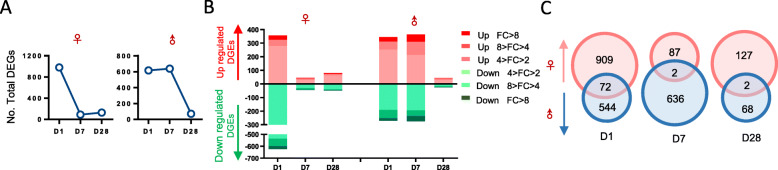
General functional profiling of the DEGs (*P* < 0.05) whose expression was significantly different (FC > 2 or < 0.5) between the IUGR and NBW piglets among the female and male piglets. **a** The total number of DEGs on Days 1 (D1), 7 (D7), and 28 (D28) among the female and male IUGR piglets. **b** Distribution of DEGs with different fold changes on D1, D7, and D28 among the female and male IUGR piglets. Different fold changes are represented by different colors. The number of DEGs from each subcategory is indicated on the right. **c** Venn diagrams of sex-specific changes in the female and male IUGR piglets on D1, D7, and D28, respectively

Given the sex-biased growth phenotypes that we observed, it was hypothesized that the transcriptomic changes also exhibited sexual dimorphic patterns in the IUGR piglet livers. Transcriptional information was analyzed between the IUGR and NBW groups within the male and female piglets (Supplementary file: Table S4-S9). Sex-specific profiling of the DGEs during postnatal development revealed different dynamics between the male and female IGUR piglets. In female IUGR piglets, the number of DGEs decreased as early as Day 7, whereas the number of DGEs decreased until Day 28 in the male IUGR piglets (Fig. 5a and b). The different patterns of gene expression raise the possibility that female IUGR piglets may have a greater potential to compensate for postnatal growth.

Secondly, we filtered sex-specific DEGs at each time point using a Venn diagram of DEGs from both female and male IUGR piglets (Fig. 5c). On Day 1, 909 DGEs were specifically dysregulated among the female IUGR piglets, whereas 544 DGEs were specifically regulated in the male IUGR piglets, and only 72 DGEs were common to both the male and female IUGR piglets. On Day 7, there were 87 and 636 DGEs specifically dysregulated in both female and male IUGR piglets, respectively, with only 2 shared DEGs between the males and females. On Day 28, 127 and 68 DGEs were specifically dysregulated in female and male IUGR piglets, with only 2 shared DEGs between the males and females. Given that the great majority of the dysregulated DEGs exhibited sexual dimorphism, we propose that the mechanisms underlying the IUGR-associated liver disorders may differ between male and female piglets.

**Fig. 6:**
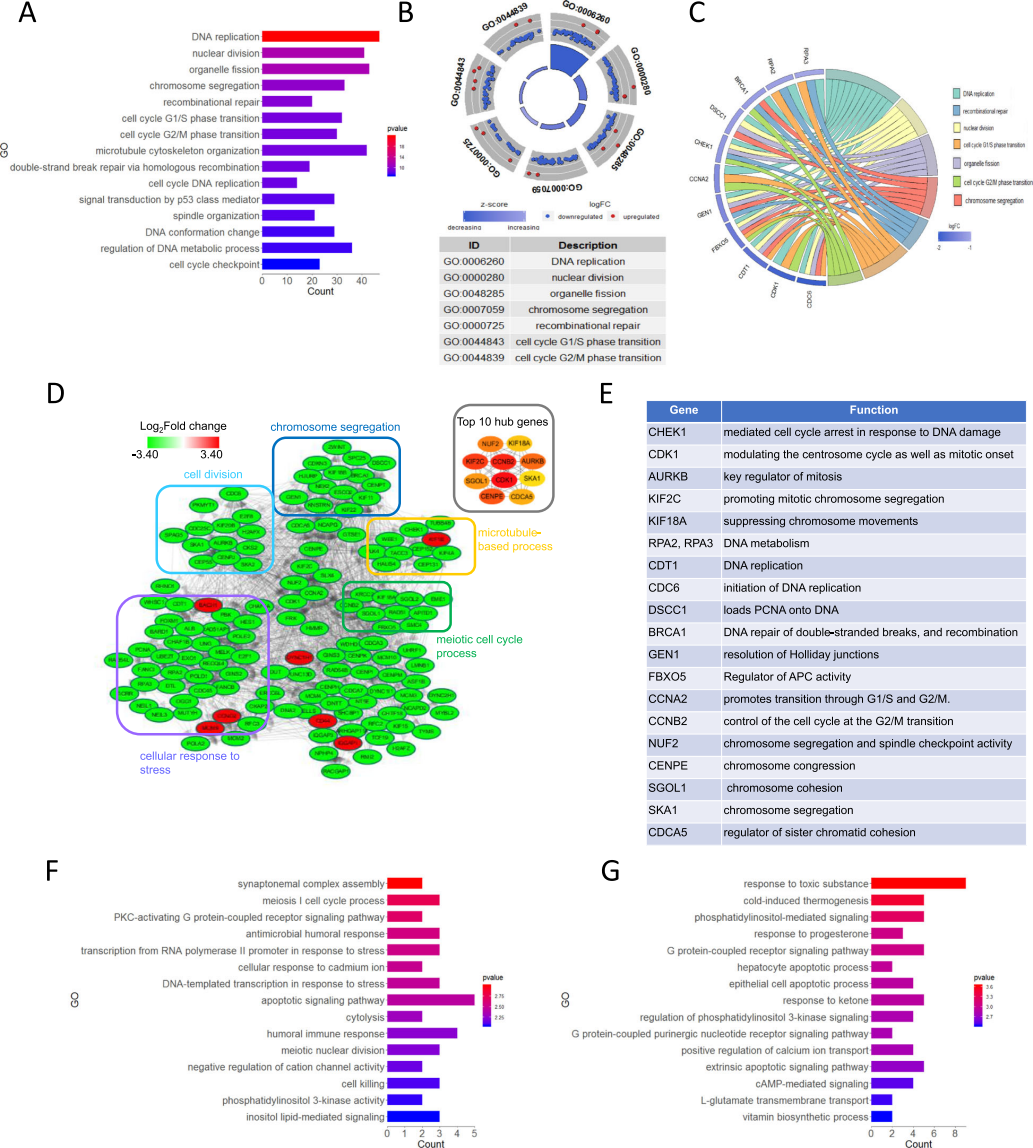
Detailed functional profiling of the DEGs (P < 0.05, FC > 2 or < 0.5) between the female IUGR and female NBW piglets. **a** Classification of the GO terms based on the functional annotation of BP enriched in the female IUGR piglets on Day 1. The ordinate represents the GO item, the abscissa represents the number of enriched DEGs corresponding to each term and the color column represents the enrichment score (defined as -Log10 *P*-value). **b** The GOCircle plot of the female IUGR piglets on Day 1. The outer circle shows a scatter plot for each term of the logFC of the assigned genes. Red circles display the up-regulated genes and the blue circles indicate the down-regulated genes by default. **c** The GOChord plot of female IUGR piglets on Day 1, and the DEGs that were assigned to at least three process terms were selected. **d** The protein-protein interaction network of the DEGs in female IUGR piglets on Day 1. The red nodes indicate the upregulated genes and the green nodes indicate the downregulated genes in the IUGR piglets. Fold-changes (FC) in gene expression are expressed as log2 (FC) values. **e** The tables show the major functions of the DEGs that were selected in the female IUGR piglets on Day 1. **f** GO enrichment analysis of the DEGs of BP enriched in female IUGR piglets on Day 7. **g** GO enrichment analysis of the DEGs of BP enriched in female IUGR piglets on Day 28

Next, to explore the possible differential mechanisms, DEGs specific to males and females were analyzed. On Day 1, the GO classification showed that the DEGs in the female IUGR were most enriched during the process of cell cycle regulation (Fig. 6a). The GOCircle plot analysis showed that most DEGs enriched in cell cycle regulation were down-regulated (Fig. 6b). With the same setting parameters as described above, the candidate DEGs were focused through the GOChord plot analysis and PPI analysis (Fig. 6c and d), including serine/threonine kinase subfamily members (*CDK1*, *AURKB*, and *CHEK1*), kinesin family members (*KIF2C* and *KIF18A*), chromosome replication, repairment-related genes (*RPA2*, *RPA3*, *CDT1*, *CDC6*, *DSCC1*, *BRCA1*, *GEN1*, and *FBXO5*), and cell cycle regulation-related genes (*CCNA1*, *CCNB2*, *NUF2*, *CENPE*, *SGOL1*, *SKA1*, and *CDDA5*). The detailed functions of these genes are presented in Fig. 6e. Similarly, the DEGs on Day 7 were also associated with the regulation of the cell cycle (e.g., ‘synaptonemal complex assembly’, ‘meiosis I cell cycle process’, and ‘meiotic nuclear division’). The candidate genes included in these terms were *STAG3*, *C11orf80*, and *SYCP2*. In addition, some physiological metabolic processes, including the regulation of transcription (*EGLN3* and *MT3*), immune response (*GNLY*, *LYZ*, and *PGC*), and apoptosis-related processes (*EPO*, *GZMB*, *IL20RA*, and *MMP9*) were also significantly enriched (Fig. 6f). On Day 28, the DEGs specific to female IUGR piglets were functionally associated with GO terms, including a response to a toxic substance (*CBL*, *HP*, and *FOS*), cold-induced thermogenesis (*ADRB1*, *NPR3*, and *PEMT*), epithelial cell apoptotic process (*ANGPT1*, *CCL2*, *KRT18,* and *KRT8*), as well as phosphatidylinositol 3-kinase signaling (*ANGPT1*, *IER3*, and *PRR5*) (Fig. 6g).

**Fig. 7:**
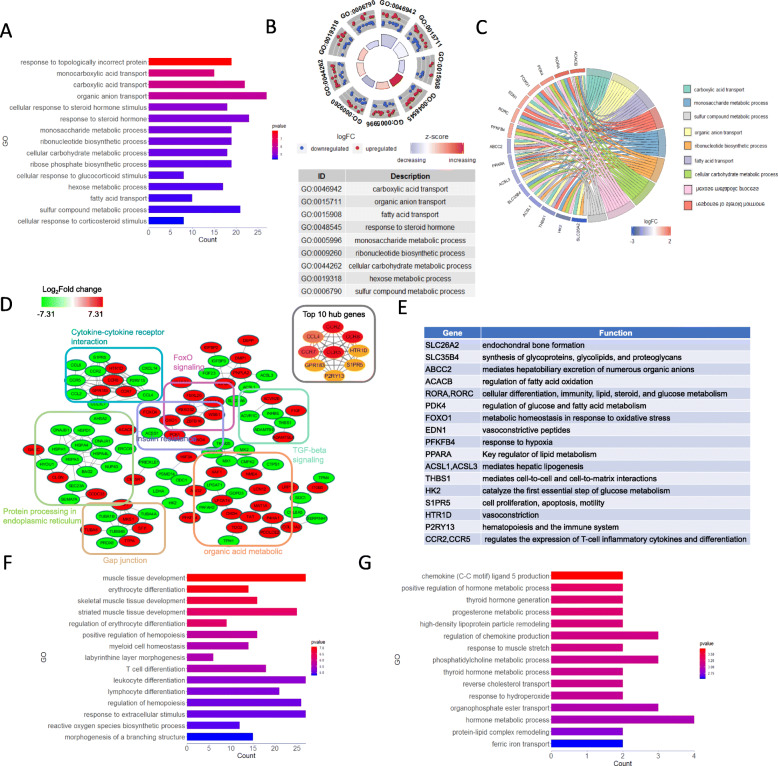
Detailed functional profiling of DEGs (P < 0.05, FC > 2 or < 0.5) between the male IUGR and male NBW piglets. **a** Classification of the GO terms based on the functional annotation of BP enriched in male IUGR piglets on Day 1. The ordinate represents the GO item, the abscissa represents the number of enriched DEGs corresponding to each term, and the color column represents the enrichment score (defined as -Log10 P-value). **b** The GOCircle plot of the male IUGR piglets on Day 1. The outer circle shows a scatter plot for each term of the logFC of the assigned genes. Red circles display the up-regulation and the blue circles indicate down-regulation by default. **c** The GOChord plot of male IUGR piglets on Day 1 and the DEGs that were assigned to at least three process terms were selected. **d** The protein-protein interaction network of the DEGs in male IUGR piglets on Day 1. The red nodes indicate upregulation and the green nodes indicate downregulation in IUGR piglets. Fold changes (FC) in expression are expressed as log2 (FC) values. **e** The tables show the major functions of the DEGs that were selected in the male IUGR piglets on Day 1. **f** The GO enrichment analysis of the DEGs of BP enriched in the male IUGR piglets on Day 7. **g** GO enrichment analysis of the DEGs of BP enriched in the male IUGR piglets on Day 28

Unlike the female IUGR group, the GO classification of DEGs in male IUGR piglets on Day 1 were enriched in factors relevant to carboxylic acid and organic anion transport, monosaccharide metabolic processes, ribonucleotide biosynthetic processes, ribose phosphate biosynthetic processes, and hexose metabolism (Fig. 7a). Using a GOCircle plot analysis, we found that most DEGs associated with transport-related processes were up-regulated, whereas the DEGs in metabolism-related processes were down-regulated (Fig. 7b). We further obtained the following candidate genes through a GOChord plot analysis, including transporters (*SLC26A2*, *SLC35B4*, and *ABCC2*) and regulatory factors involved in both glucose and lipid metabolism (*RORA*, *RORC*, *PDK4*, *PPARA*, *ACSL1*, *ACSL3*, and *HK2*) (Fig. 7c). In addition, CytoHubba revealed that most of the top 10 hub genes were cytokines (*CCL4*, *CCR5*, *CCR7*, *CCR8*, *CCR2*, and *GPR183*) (Fig. 7d and e), which were involved in inflammatory regulation, indicating an immune response disorder in male IUGR piglets. Next, we analyzed the GO cluster on Day 7, DEGs of the male IUGRs were enriched regarding immune cell differentiation (*SOCS1*, *NFKBIZ*, and *LAG3*) and hematopoiesis (*GATA1*, *MYB*, *TAL1*, *TRIM58*, *HLX*, *DLL1*, and *MAFB*) (Fig. 7f). In the end, the GO items in the male IUGR on Day 28 included thyroid hormone metabolic processes (*DIO2*, *PAX8*, *EGR1*, and *AFP*) and lipoprotein transport (*APOA4*, *MFSD2A*, *LIPG*, *SLC25A33*, and *SLC44A5*). (Fig. 7g).

### Serum lipid metabolites between the IUGR and NBW piglets

**Fig. 8:**
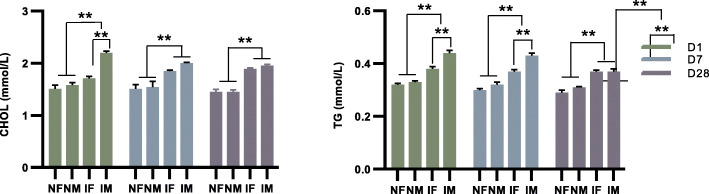
Serum lipid metabolites between IUGR and NBW piglets. The serum level of CHOL and TG in IUGR piglets compared with NBW (*P < 0.05; ** *P* < 0.01). NF, female NBW; NM, male NBW; IF, female IUGR; IM, male IUGR

To explore the metabolic status of IUGR livers, we next tested the serum lipid profiles, highlighting the cholesterol (CHOL) and triglycerides (TG) that were commonly used as the clinical indexes to reflect the physiological or pathological state of the liver. As the results showed in Fig. 8, the level of CHOL and TG were both significantly increased in the IUGR piglets compared with the NBW groups for both the female and male groups. Interestingly, the TG level of male IUGR piglets was consistently higher than that of the female IUGR piglets from Days 1 to 7, and the CHOL level of male IUGR piglets was also significantly higher than that of female IUGR piglets on Day 1, which also exhibited sexual dimorphic patterns.

## Discussion

Body weight and ADG are typically used as important indicators for piglet growth performance. The present study revealed that growth compensation occurred in newborn IUGR piglets, which is consistent with the previous findings that IUGR individuals have a potential compensatory mechanism for relatively rapid weight gain [4, 15]. Although catch-up growth was considered to be a positive benefit for growth improvement [16], increasing evidence indicates that there are important metabolic disorders in IUGR neonates. In addition, postnatal catch-up growth in IUGR neonates appears to be associated with increased fat mass rather than lean mass, and may progress to insulin resistance and altered glucose homeostasis [17], both of which are thought to contribute to a higher risk for overweight/obesity and other related short- and long-term health complications (e.g., poor physical growth, metabolic syndrome, cardiovascular disease, neurodevelopmental impairment, and endocrine abnormalities) [18, 19]. To the best of our knowledge, this study is the first comprehensive profiling of the liver transcriptome using postnatal IUGR piglets as a model, providing a reference for balancing growth compensation and preventing complications.

### Dysregulation in immune function

We found that several constantly dysregulated DEGs in IUGR piglets were involved in the immune response, including ‘lymphocyte migration’, ‘leukocyte cell-cell adhesion’, ‘regulation of chemotaxis’, and ‘regulation of leukocyte activation’ on Day 1, as well as ‘positive regulation of T cell proliferation’, ‘innate immune response-activating signal transduction’, and ‘type I interferon biosynthetic process’ on Day 28. The liver has been identified as a key, frontline immune tissue, and an anti-inflammatory or immunotolerant status has been found to represent the baseline immune environment of the liver. Moreover, the balance between immunity and tolerance is essential to ensure appropriate liver function [20]. The recruitment and activation of leukocytes and lymphocytes suggests the presence of both hepatic injury and inflammation. In addition, enriched DGEs contain both pro- and anti-inflammatory cytokines (e.g., *TNF*, *CCL2*, and *CCL4*), which are mediated and regulated by the immune system in the liver. Various pathological receptors involved in modulating the immune response (e.g., *TLR8*, *GPR183*, *CD209*, *CCR7*, and *CCR8*) were also identified, which suggests that the liver of IUGR piglets may suffer from dysregulated immune function.

In addition, the plasma aminotransferases (ALT and AST) have been widely used to detect liver function due to their high sensitivity. These enzymes were normally predominantly contained within the cytoplasm and mitochondria of hepatocytes; however, when the liver becomes injured or damaged under pathological conditions, the liver cells release these enzymes into the blood, raising the levels of AST and ALT enzymes in the blood, signaling liver disease [21]. Therefore, a higher serum activity of ALT and AST could be an indicator of liver inflammation in IUGR piglets. Moreover, the total protein is another important indicator of the metabolic capacity of the liver [22], which was significantly lower in the IUGR piglets. This finding indicates the presence of liver damage and dysfunction in IUGR piglets. The level of damage and inflammatory stress was also supported by the degree of lymphocytic infiltration and vacuolization in the hepatic lobules of IUGR piglets.

### Disorganized cytoskeleton and membrane structure

We also found that IUGR piglet livers were associated with cytoskeletal disorganization. The cytoskeleton maintains the cellular shape, organizes and tethers the organelles, and plays an important role in molecule transport, locomotion, and cell signaling [23]. Moreover, actin filaments have been found to be fundamental for cellular motility and morphogenic processes, including migration, ion channel activity, secretion, apoptosis, and cell survival [24]. It has been confirmed that an altered cytoskeletal organization was correlated with the changes in the cell shape and consequently mediated cellular transformation in human liver cells [25, 26]. In our study, the presence of DEGs (e.g., *ADD2*, *KIAA1211*, and *SPTB*) involved in providing architectural and functional support of cell morphology, combined with ultrastructural cytoskeleton deformation in the hepatocytes of IUGR piglets (e.g., loss of cytoplasmic material, swollen mitochondria, dilatation, and discontinuity in the endoplasmic reticulum), suggest that the dynamic process of polymerization or depolymerization of actin filaments was highly disorganized in the IUGR piglets.

### Transcriptional dysregulation

In our study, DEGs related to transcriptional factors (e.g., *EGR1*, *EGR2*, *SHOX2*, and *FOS*) were clustered in the IUGR piglets. Numerous studies have established that transcription factors play a pivotal role in liver development and cellular functionality, which provides solid evidence for the coherence and synergy of various transcription factors on liver-specific gene expression [27]. Transcription factors form a network with coactivators, corepressors, enzymes, DNA, and RNA to either repress or activate liver-specific gene expression [28]. Thus, these findings suggest that transcriptional dysregulation can lead to altered intermolecular interactions with the consequence of liver dysfunction in IUGR piglets.

### Glucocorticoids participate in IUGR liver metabolism

DEGs related to glucocorticoid and steroid hormone regulation were also observed in IUGR piglet livers. A number of reports have indicated that children with IUGR may have a higher ratio of cortisol to cortisone, which may permanently influence adrenal function due to reprogramming of the hypothalamic-pituitary-adrenal axis caused by intrauterine malnutrition or chronic stress [29]. The primary role of glucocorticoids in the liver is to regulate hepatic energy homeostasis and control hepatic influx and the efflux of lipids [30, 31]. Moreover, both liver steatosis and fibrosis have been shown to be promoted by substantial glucocorticoid exposure and receptor antagonism [32]. In addition, glucocorticoids were found to be potent modulators of the IGF system, as they were able to reduce the local expression of *IGF1* [33]. We found that several DEGs are related to glucocorticoid functions (e.g., *IGF1*, *IGFBP2*, *AGTR2*, and *NTRK3*). The observation of downregulated *IGF1* was consistent with the previous report of a deficient IGF1 signaling pathway in IUGR rats [34].

### Sexual dimorphism in response to IUGR

Our study found a sex-biased developmental dynamic between male and female IUGR piglets. DEGs in the female IUGR livers are mainly associated with cell cycle progression, which may explain the more evident growth compensation of the female IUGR piglets. In contrast, the male IUGR piglet livers were characterized by hepatic transporter dysregulation (e.g., organic anion and monocarboxylate transporter). Transporters have been identified and characterized as important determinants for nutrition absorption, distribution, metabolism, and excretion in organ development and cellular function [35, 36]. Organic anion transporters are abundantly expressed in the liver and promote the uptake of various endogenous substrates, including bile acids and various exogenous drugs [37]. Monocarboxylate transporters have been widely studied due to their importance in the transporting L-lactate, pyruvate, and fatty acids across the plasma membrane [38]. Interestingly, the expression of hepatic transporters varies in a sex-dependent manner between female and male IUGR piglets during development. Similar notions were also supported by the fact that the expression of organic anion transporters also exhibited sexual dimorphic patterns in both male and female rats [39].

In addition, increasing evidence has demonstrated that male IUGR individuals are more sensitive to the IUGR-associated physiological and pathological changes than females [40, 41]. Thus, it is proposed that male offspring have a survival disadvantage compared to their female littermates [42]. For example, male offspring have a higher risk of developing cardiovascular and metabolic diseases later in life [43]. Here, the same phenomenon was observed in our study, with a higher level of cholesterol and triglycerides associated with the male IUGR piglets. Based on the observations of the sexual-dimorphic effects on the serum lipid metabolites between the IUGR and NBW piglets, male IUGR piglets appear to be more sensitive to a disruption of metabolic homeostasis.

## Conclusion

In summary, our study demonstrates that the livers of IUGR piglets were associated with a series of complications, including inflammatory stress and immune function dysregulation; disorganized cytoskeleton and membrane structure; transcriptional dysregulation; and abnormal glucocorticoid metabolism. These observations provide a detailed reference for understanding the mechanisms and characterization of the IUGR liver functions. Thus, a promising intervention strategy should consider the balance between postnatal catch-up growth and adverse metabolic consequences in IUGR piglets. In particular, our results showcase the sex-biased developmental dynamics between male and female IUGR piglets, suggesting that potential interventions should be specific for male or female IUGR piglets.

## Materials and methods

### Data and sample collection

**Fig. 9:**
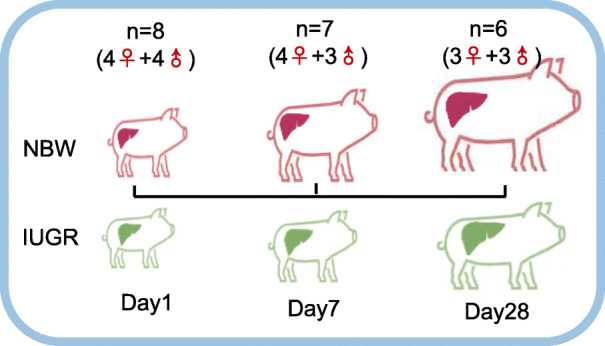
Overview of the experimental design. Based on their birth weight, the piglets were classified into either the IUGR or NBW group. There was an equal ratio of males to females. Liver tissues were collected on Days 1, 7, and 28, respectively

All the animals were obtained from the experiment pig farm of Institute of Animal Science, Chinese Academy of Agricultural Sciences (CAAS). The experimental protocols in this study were approved by the Science Research Department of the Institute of Animal Science, Chinese Academy of Agricultural Sciences (CAAS) (Beijing, China). The study design is presented in Fig. 9. The study involved approximately 50 healthy sows (Large White) from the first or second parity farrowed from the same batch, and piglets (Duroc sires × Large White dams) were spontaneously delivered at term. Newborn piglets were classified according to their birth weight as IUGR and NBW, respectively. The IUGR piglets were strictly selected based on the following well-accepted criteria: the average body weight in the litter being less than two standard deviations, whereas NBW were identified as being within one standard deviation of the mean body weight [4, 44–46]. Piglets were randomly assigned and sacrificed on Day 1 (D1), Day 7 (D7), and Day 28 (D28) in both the IUGR and NBW groups, with eight replicates (50% males and 50% females) at each of the time points in each group. The gender of the siblings was the same for each time point and from each litter in both the IUGR and NBW piglets. Due to the high early mortality rate in IUGR piglets, one piglet from the Day 7 and two piglets from the Day 28 of the IUGR group died of disease prior to the experimental endpoint. Therefore, we excluded the NBW sibling piglets in each group, and collected samples from 42 piglets [Day 1: IUGR *n* = 8 (4 females and 4 males) vs NBW n = 8 (4 females and 4 males); Day 7: IUGR *n* = 7 (4 females and 3 males) vs NBW n = 7 (4 females and 3 males); Day 28: IUGR *n* = 6 (3 females and 3 males) vs NBW n = 6 (3 females and 3 males)]. Cross-fostering was performed, except for the piglets selected for the experiments that remained with their original sow. All of the piglets were managed in accordance with the routine farm procedures. At an age of three days, the piglets were subjected to tail-docking. At the same time, piglets received an oral coccidiostat drugs and an intramuscular injection of iron dextran glucoheptonate. The birth weight and the body weight (BW) was measured at the time of sacrifice, and the average daily gain (ADG) of the periods of Day 1–7 and Day 1–28 of age was calculated [(final weight - initial weight)/number of days].

Blood samples of piglets at each of the time points were collected by an anterior vena cava puncture and centrifuged at 2000×*g* for 10 min at 4 °C. The collected serum was stored at − 80 °C for further analysis.

Based on the procedures described by previous studies [47, 48], the euthanasia of piglets was preceded by an induction of local anesthesia with an abdominal subcutaneous injection of 2% lidocaine hydrochloride (4.5 mg/kg, Shandong Hualu Pharmaceutical Co., Ltd., Shandong, China). Following a 10 min delay, the piglets were euthanized by an intraperitoneal injection of an overdose of sodium pentobarbital (100 mg/kg, Sigma, St. Louis, MO, USA), followed by jugular exsanguination.

Liver samples were collected from the left lateral lobe and washed in phosphate-buffered saline immediately. A portion of the liver segment was fixed in 4% paraformaldehyde at 4 °C for histological analysis, the other part of the liver tissue was frozen in liquid nitrogen and stored at − 80 °C until the RNA was extracted for further analysis. The right lateral lobe of the liver was prepared for transmission electron microscopy (TEM).

### RNA-Seq and biological analysis

The RNA-seq process was performed as previously described in accordance with the following steps [49]: RNA samples were isolated using TRIzol reagent (Invitrogen, Carlsbad, CA, USA), library construction was performed using a TruSeq RNA Sample Prep kit (Illumina, San Diego, CA, USA), and sequencing of the library was constructed with the Illumina HiSeq 2500 platform (Illumina). The sequencing data used in this study are available at the Sequence Read Archive, BioProject: PRJNA597972 (https://www.ncbi.nlm.nih.gov/bioproject/PRJNA597972).

The bioinformatics analysis of RNA-Seq involved reads quality control, alignment of the filtered sequence reads to the Sscrofa10.2 reference genome from the Ensembl annotation system (ftp://ftp.ensembl.org/pub/release-89/fasta/sus_scrofa/dna/), and differential expression analysis was performed by DESeq [50]. The genes with an adjusted *P*-value (*P* < 0.05) and fold-change (FC, two-fold) were assigned as a threshold to filter the differentially expressed genes (DEGs) for further biological analysis. The database used for the annotation, visualization, and integrated was DAVID v6.8 (http://david.abcc.ncifcrf.gov) and pathway analysis was performing using the Kyoto Encyclopedia of Genes and Genomes (KEGG) (http://www.genome.jp/kegg). Protein-protein interaction networks were constructed using the STRING database (v.10.5; https://string-db.org/). STRING networks were downloaded and hub nodes were identified using Cytoscape (v.3.7.1) based on the number of connections between nodes. GOplot Analyses were performed using R software version 3.6.1 [51].

### RNA isolation and quantitative by real-time qPCR

Total RNA extraction from the piglet livers was performed using TRIzol according to the manufacturer’s instructions. RNA was then used for reverse transcription using a commercially available first strand cDNA synthesis kit (iScript cDNA Synthesis Kit; Bio-Rad Laboratories, USA). Real-time qPCR was performed using the Bio-Rad CFX96 RealTime PCR System using SsoFast EvaGreen Supermix (Bio-Rad Laboratories, Hercules, CA, USA), as previously described [52]. The real-time qPCR reaction was repeated three times independently and the primers (*SLC38A5*, *SLC51B*, *DMRTA1*, *ADAD1*, and *CD200R1*) are listed in Supplementary Table S10.

### Liver histologic evaluation

The procedure for liver hematoxylin eosin (H&E) staining was performed in accordance with a previous report [53]. Liver tissue specimens from the left lobe were fixed and stained with H&E. After H&E staining, a morphologic evaluation was performed using a light microscope (Nikon ECLIPSE 80i; Nikon Corporation). A double-blind assessment was performed by two independent observers to ensure the accuracy of the liver histological evaluation, and each section was observed in at least three different fields of view.

### Liver morphological evaluation

Liver samples were prepared for transmission electron microscopy (TEM) according to the previously described procedure in [54]. The liver tissue was trimmed to less than 1 mm^3^ using a razor blade and fixed in 2.5% glutaraldehyde for 4 h, post-fixed in 1% osmium tetroxide for 2 h, dehydrated, and embedded in an epoxy resin. The tissue was sliced into ultrathin sections of 60–80 nm using an ultramicrotome (Leica EM UC7), stained with 2% uranyl acetate and lead citrate for double contrasting, and observed under a transmission electron microscope (Hitachi HT-7700).

### Assessment of the serum metabolite contents

The concentration of the total protein (TP), triglyceride (TG), cholesterol (CHOL), as well as the serum enzyme activity of alanine aminotransferase (ALT) and aspartate aminotransferase (AST) was detected with a Hitachi automatic biochemical analyzer (7600–120, Hitachi Ltd., Tokyo, Japan).

### Statistical analysis

Statistical analysis was performed using the general linear mixed model (GLMM) procedure in SAS version 9.4 (Statistical Analysis System Institute Inc., USA). All variables were assessed with a least square analysis (LSA) comparison between the IUGR and NBW experimental groups. Group (LBW/NBW) and gender (female/male) were included in the model as the fixed effects, and interactions between the group and gender were examined. All of the results were expressed as least squares means (lsmeans) ± standard error (SE), and a *P* value was presented to indicate a statistical significance between the IUGR and NBW groups. Statistical significance was considered to be significant (*P* < 0.05) and extremely significant (*P* < 0.01).

## Supplementary information

**Additional file 1: Table S1.** DEGs between the IUGR and NBW piglets on day 1 (Fold change > 2 or < 0.5, *P* < 0.05). **Table S2.** DEGs between the IUGR and NBW piglets on day 7 (Fold change > 2 or < 0.5, P < 0.05). **Table S3.** DEGs between the IUGR and NBW piglets on day 28 (Fold change > 2 or < 0.5, P < 0.05). **Table S4.** DEGs between the female IUGR and female NBW piglets on day 1 (Fold change > 2 or < 0.5, P < 0.05). **Table S5.** DEGs between the female IUGR and female NBW piglets on day 7 (Fold change > 2 or < 0.5, P < 0.05). **Table S6.** DEGs between the female IUGR and female NBW piglets on day 28 (Fold change > 2 or < 0.5, P < 0.05). **Table S7.** DEGs between the male IUGR and male NBW piglets on day 1 (Fold change > 2 or < 0.5, P < 0.05). **Table S8.** DEGs between the male IUGR and male NBW piglets on day 7 (Fold change > 2 or < 0.5, P < 0.05). **Table S9.** DEGs between the male IUGR and male NBW piglets on day 28 (Fold change > 2 or < 0.5, P < 0.05). **Table S10.** Primer sequences of the differentially expressed genes identified by quantitative real-time polymerase chain reaction.

## Data Availability

The datasets analyzed are available from the Sscrofa10.2 reference genome downloaded from the Ensembl website (ftp://ftp.ensembl.org/pub/release-89/fasta/sus_scrofa/dna/) and all data generated or analyzed during the current study are included in this published article and its supplementary information files. The sequencing data used in this study are available at the Sequence Read Archive, BioProject: PRJNA597972 (https://www.ncbi.nlm.nih.gov/bioproject/PRJNA597972).

## Abbreviations

IUGR: intrauterine growth restriction
NBW: normal body weight
BW: body weight
ADG: average daily gain
DEGs: differentially expressed genes
GO: Gene Ontology
KEGG: Kyoto Encyclopedia of Genes and Genomes
BP: biological processes
PPI: protein–protein interaction
ALT: alanine aminotransferase
AST: aspartate aminotransferase
TP: total protein
TG: triglyceride
CHOL: cholesterol

## References

[1] Wu G, Bazer FW, Wallace JM, Spencer TE (2006). Board-invited review: intrauterine growth retardation: implications for the animal sciences. J Anim Sci.

[2] Wang X, Zhu Y, Feng C, Lin G, Wu G, Li D, Wang J (2018). Innate differences and colostrum-induced alterations of jejunal mucosal proteins in piglets with intra-uterine growth restriction. Br J Nutr.

[3] Zhang H, Li Y, Hou X, Zhang L, Wang T (2016). Medium-chain TAG improve energy metabolism and mitochondrial biogenesis in the liver of intra-uterine growth-retarded and normal-birth-weight weanling piglets. Br J Nutr.

[4] Li W, Li B, Lv J, Dong L, Zhang L, Wang T (2018). Choline supplementation improves the lipid metabolism of intrauterine-growth-restricted pigs. Asian-Australas J Anim Sci.

[5] Zheng P, Song Y, Tian Y, Zhang H, Yu B, He J, Mao X, Yu J, Luo Y, Luo J (2018). Dietary arginine supplementation affects intestinal function by enhancing antioxidant capacity of a nitric oxide-independent pathway in low-birth-weight piglets. J Nutr.

[6] Feng C, Bai K, Wang A, Ge X, Zhao Y, Zhang L, Wang T (2018). Effects of dimethylglycine sodium salt supplementation on growth performance, hepatic antioxidant capacity, and mitochondria-related gene expression in weanling piglets born with low birth weight. J Anim Sci.

[7] Peterside IE, Selak MA, Simmons RA (2003). Impaired oxidative phosphorylation in hepatic mitochondria in growth-retarded rats. Am J Physiol Endocrinol Metab.

[8] Lane RH, Flozak AS, Ogata ES, Bell GI, Simmons RA (1996). Altered hepatic gene expression of enzymes involved in energy metabolism in the growth-retarded fetal rat. Pediatr Res.

[9] Su W, Xu W, Zhang H, Ying Z, Zhou L, Zhang L, Wang T (2017). Effects of dietary leucine supplementation on the hepatic mitochondrial biogenesis and energy metabolism in normal birth weight and intrauterine growth-retarded weanling piglets. Nutr Res Pract.

[10] Limesand SW, Jensen J, Hutton JC, Hay WW (2005). Diminished beta-cell replication contributes to reduced beta-cell mass in fetal sheep with intrauterine growth restriction. Am J Physiol Regul Integr Comp Physiol.

[11] Thorn SR, Regnault TR, Brown LD, Rozance PJ, Keng J, Roper M, Wilkening RB, Hay WW, Friedman JE (2009). Intrauterine growth restriction increases fetal hepatic gluconeogenic capacity and reduces messenger ribonucleic acid translation initiation and nutrient sensing in fetal liver and skeletal muscle. Endocrinology..

[12] Xing K, Zhu F, Zhai L, Liu H, Wang Z, Hou Z, Wang C (2014). The liver transcriptome of two full-sibling Songliao black pigs with extreme differences in backfat thickness. J Anim Sci Biotechnol.

[13] Ren W, Badgery W, Ding Y, Guo H, Gao Y, Zhang J (2019). Hepatic transcriptome profile of sheep (Ovis aries) in response to overgrazing: novel genes and pathways revealed. BMC Genet.

[14] Xia J, Yuan J, Xin L, Zhang Y, Kong S, Chen Y, Yang S, Li K (2014). Transcriptome analysis on the inflammatory cell infiltration of nonalcoholic steatohepatitis in bama minipigs induced by a long-term high-fat, high-sucrose diet. PLoS One.

[15] Ziegler EE (2015). Nutrient needs for catch-up growth in low-Birthweight infants. Nestle Nutr Inst Workshop Ser.

[16] Tang A, Slopen N, Nelson CA, Zeanah CH, Georgieff MK, Fox NA (2018). Catch-up growth, metabolic, and cardiovascular risk in post-institutionalized Romanian adolescents. Pediatr Res.

[17] Cho WK, Suh BK (2016). Catch-up growth and catch-up fat in children born small for gestational age. Korean J Pediatr.

[18] Kesavan K, Devaskar SU (2019). Intrauterine growth Restriction: postnatal monitoring and outcomes. Pediatr Clin N Am.

[19] Devaskar SU, Chu A (2016). Intrauterine Growth Restriction: Hungry for an answer. Physiology (Bethesda).

[20] Kubes P, Jenne C (2018). Immune responses in the liver. Annu Rev Immunol.

[21] McGill MR (2016). The past and present of serum aminotransferases and the future of liver injury biomarkers. EXCLI J.

[22] Tóthová C, Mihajlovičová X, Nagy O (2018). The Use of Serum Proteins in the Laboratory Diagnosis of Health Disorders in Ruminants.

[23] Fletcher DA, Mullins RD (2010). Cell mechanics and the cytoskeleton. Nature..

[24] Papakonstanti EA, Stournaras C (2008). Cell responses regulated by early reorganization of actin cytoskeleton. FEBS Lett.

[25] Liu YH, Cheng CC, Ho CC, Chao WT, Pei RJ, Hsu YH, Ho LC, Shiu BH, Lai YS (2011). Plectin deficiency on cytoskeletal disorganization and transformation of human liver cells in vitro. Med Mol Morphol.

[26] Cheng CC, Lai YC, Lai YS, Chao WT, Tseng YH, Hsu YH, Chen YY, Liu YH (2016). Cell Pleomorphism and cytoskeleton disorganization in human liver cancer. In Vivo.

[27] Schrem H, Klempnauer J, Borlak J (2002). Liver-enriched transcription factors in liver function and development. Part I: the hepatocyte nuclear factor network and liver-specific gene expression. Pharmacol Rev.

[28] Schrem H, Klempnauer J, Borlak J (2004). Liver-enriched transcription factors in liver function and development. Part II: the C/EBPs and D site-binding protein in cell cycle control, carcinogenesis, circadian gene regulation, liver regeneration, apoptosis, and liver-specific gene regulation. Pharmacol Rev.

[29] Cianfarani S, Geremia C, Scott CD, Germani D (2002). Growth, IGF system, and cortisol in children with intrauterine growth retardation: is catch-up growth affected by reprogramming of the hypothalamic-pituitary-adrenal axis?. Pediatr Res.

[30] Yu CY, Mayba O, Lee JV, Tran J, Harris C, Speed TP, Wang JC (2010). Genome-wide analysis of glucocorticoid receptor binding regions in adipocytes reveal gene network involved in triglyceride homeostasis. PLoS One.

[31] Vegiopoulos A, Herzig S (2007). Glucocorticoids, metabolism and metabolic diseases. Mol Cell Endocrinol.

[32] Koorneef LL, van den Heuvel JK, Kroon J, Boon MR, t’Hoen PAC, Hettne KM, van de Velde NM, Kolenbrander KB, TCM S, Mol IM (2018). Selective glucocorticoid receptor modulation prevents and reverses nonalcoholic fatty liver disease in male mice. Endocrinology..

[33] Cianfarani S, Ladaki C, Geremia C (2006). Hormonal regulation of postnatal growth in children born small for gestational age. Horm Res.

[34] He Z, Zhang J, Huang H, Yuan C, Zhu C, Magdalou J, Wang H (2019). Glucocorticoid-activation system mediated glucocorticoid-insulin-like growth factor 1 (GC-IGF1) axis programming alteration of adrenal dysfunction induced by prenatal caffeine exposure. Toxicol Lett.

[35] Stieger B, Hagenbuch B (2016). Recent advances in understanding hepatic drug transport. F1000Res.

[36] Shugarts S, Benet LZ (2009). The role of transporters in the pharmacokinetics of orally administered drugs. Pharm Res.

[37] Li TT, An JX, Xu JY, Tuo BG (2019). Overview of organic anion transporters and organic anion transporter polypeptides and their roles in the liver. World J Clin Cases.

[38] Jones RS, Morris ME (2016). Monocarboxylate transporters: therapeutic targets and prognostic factors in disease. Clin Pharmacol Ther.

[39] Brzica H, Abdullahi W, Reilly BG, Ronaldson PT (2018). Sex-specific differences in organic anion transporting polypeptide 1a4 (Oatp1a4) functional expression at the blood-brain barrier in Sprague-Dawley rats. Fluids Barriers CNS.

[40] Alur P (2019). Sex differences in nutrition, growth, and metabolism in preterm infants. Front Pediatr.

[41] Aiken CE, Ozanne SE (2013). Sex differences in developmental programming models. Reproduction..

[42] Muralimanoharan S, Li C, Nakayasu ES, Casey CP, Nathanielsz PW, Maloyan A, Metz TO (2017). Sexual dimorphism in the fetal cardiac response to maternal nutrient restriction. J Mol Cell Cardiol.

[43] Dasinger JH, Alexander BT (2016). Gender differences in developmental programming of cardiovascular diseases. Clin Sci (Lond).

[44] Wang X, Lin G, Liu C, Feng C, Zhou H, Wang T, Li D, Wu G, Wang J (2014). Temporal proteomic analysis reveals defects in small-intestinal development of porcine fetuses with intrauterine growth restriction. J Nutr Biochem.

[45] Chen F, Wang T, Feng C, Lin G, Zhu Y, Wu G, Johnson G, Wang J (2015). Proteome differences in placenta and endometrium between Normal and intrauterine growth restricted pig fetuses. PLoS One.

[46] Paredes SP, Jansman AJ, Verstegen MW, den Hartog LA, van Hees HM, Bolhuis JE, van Kempen TA, Gerrits WJ (2014). Identifying the limitations for growth in low performing piglets from birth until 10 weeks of age. Animal.

[47] Poletto R, Steibel JP, Siegford JM, Zanella AJ (2006). Effects of early weaning and social isolation on the expression of glucocorticoid and mineralocorticoid receptor and 11beta-hydroxysteroid dehydrogenase 1 and 2 mRNAs in the frontal cortex and hippocampus of piglets. Brain Res.

[48] Hedemann MS, Dybkjær L, Jensen BB (2007). Pre-weaning eating activity and morphological parameters in the small and large intestine of piglets. Livest Sci.

[49] Kwon SG, Hwang JH, Park DH, Kim TW, Kang DG, Kang KH, Kim IS, Park HC, Na CS, Ha J, Kim CW (2016). Identification of differentially expressed genes associated with litter size in Berkshire pig placenta. PLoS One.

[50] Anders S, Huber W (2010). Differential expression analysis for sequence count data. Genome Biol.

[51] Walter W, Sanchez-Cabo F, Ricote M (2015). GOplot: an R package for visually combining expression data with functional analysis. Bioinformatics.

[52] Ren L, Zhang C, Tao L, Hao J, Tan K, Miao K, Yu Y, Sui L, Wu Z, Tian J, An L (2017). High-resolution profiles of gene expression and DNA methylation highlight mitochondrial modifications during early embryonic development. J Reprod Dev.

[53] He J, Niu Y, Wang F, Wang C, Cui T, Bai K, Zhang J, Zhong X, Zhang L, Wang T (2018). Dietary curcumin supplementation attenuates inflammation, hepatic injury and oxidative damage in a rat model of intra-uterine growth retardation. Br J Nutr.

[54] Du J, Zhang X, Han J, Man K, Zhang Y, Chu ES, Nan Y, Yu J (2017). Pro-inflammatory CXCR3 impairs mitochondrial function in experimental non-alcoholic Steatohepatitis. Theranostics..

